# Impact of 2% carbon dioxide during storage on egg quality and early embryo development

**DOI:** 10.1016/j.psj.2026.107466

**Published:** 2026-07-17

**Authors:** P. Javaloyes, N. Bernardet, F. Elleboudt, H. Adriaensen, A. Collin, S. Réhault-Godbert

**Affiliations:** a INRAE, Université de Tours, BOA, Nouzilly, France; b INRAE, Université de Tours, CHU de Tours, PIXANIM, 37380, Nouzilly, France

**Keywords:** Storage, CO_2_, Egg quality, Blastoderm, Embryo

## Abstract

Storing fertilized eggs for several weeks prior to incubation is a hatchery practice to meet fluctuating demands for chicks. However, prolonged storage leads to a progressive deterioration of the egg internal structures, which ultimately reduces hatchability. This decrease in egg quality results from an increase in albumen pH following the diffusion of CO_2_ naturally present in the egg, through the eggshell pores. In this study, we explored whether a CO_2_-enriched atmosphere (2%) could limit CO_2_ diffusion from eggs stored for 6, 13, 20, or 27 days at 12°C and 80% relative humidity, followed by a 24-hour acclimatization at 20°C (as commonly practiced in hatcheries prior to incubation). Egg internal quality and selected blastoderm characteristics (position relative to the air cell and diameter) were evaluated using a combination of classical measurements and non-invasive imaging techniques (computed tomography and magnetic resonance imaging). A subset of eggs was incubated for seven days to evaluate embryo viability and mass.

The principal component analysis revealed a clear separation between eggs stored with CO_2_ and those in the control group. While storage duration negatively affected most parameters, CO_2_ was shown to partly mitigate egg dehydration (as indicated by lower mass loss and air cell volume) and the progressive degradation of albumen and yolk index over time, compared with control eggs stored without CO_2_. However, the size of the blastoderm was reduced significantly in CO_2_ eggs following prolonged storage, compared with control eggs. This result suggests that 2% CO_2_ may alter the viability of blastoderm cells. Nevertheless, no impact on the viability of embryos incubated for seven days was observed in CO_2_ eggs, although they exhibited a significant decrease in embryo mass compared with control eggs.

Collectively, these findings support the hypothesis that 2% CO_2_ during storage prior to incubation partly prevents the degradation of internal egg components over time, but the potential benefits of this storage practice on embryonic development and growth during the entire incubation period require further investigation.

## Introduction

The storage of fertilized eggs prior to incubation is a common practice in commercial hatcheries. During storage, eggs undergo diapause characterized by low metabolic activity and an arrest of blastoderm cell division at the G2 phase of the cell cycle (Ko et al., 2017). This pause is triggered by exposure of eggs to temperatures below the physiological zero (approximately 21°C (Fasenko, 2007)). However, diapause does not systematically guarantee the normal resumption of embryonic development upon incubation. Indeed, the temperature of storage interacts with relative humidity, storage duration, and egg orientation, which collectively influence embryonic survival during both storage and incubation (Bakst and Akuffo, 2002). As storage duration increases, hatchability declines (Byng and Nash, 1962; Fasenko, et al., 1992), while chick quality is adversely affected (Tona, et al., 2003, 2004). To optimize egg quality, the storage temperature must be adjusted according to the duration of storage. In practice, it is recommended to store eggs at a maximum temperature of 18°C for short-term storage and at approximately 12°C for storage exceeding 15 days (Proudfoot, et al., 1990). This recommendation is supported by a study demonstrating that the cytoarchitecture of blastoderms stored at 18°C is reorganized due to cellular mitosis, which negatively impacts the resumption of development after diapause. In contrast, the low metabolic activity in mitotically arrested cells observed in eggs stored at 12°C appears to protect cells from death, thereby allowing for better embryonic survival after prolonged storage (Pokhrel et al., 2018).

The alteration of internal egg quality is characterized by progressive dehydration leading to weight loss, an increase in albumen pH resulting in its thinning, deterioration of the vitelline membrane surrounding the yolk, and damage to embryonic cells within the blastoderm (Adriaensen, et al., 2022; Brady, et al., 2022). The diffusion of internal gases, namely water and CO_2_, through eggshell pores, is assumed to be responsible for this decline in egg quality. Mueller (1958) showed that the albumen of a freshly laid egg contains approximately 55 mg of CO_2_, which diffuses rapidly at the beginning of storage (Mueller, 1958). The CO_2_ flux depends on temperature, with a loss of approximately 9.0 mg/day at the start of storage, decreasing to about 0.8 mg/day after four days at 30°C or after twelve days at 12°C. This progressive loss of CO_2_ leads to an alkalinization of albumen, with pH increasing from approximately 7.8 in freshly laid eggs to a maximum of 9.5 in stored eggs (Mueller, 1958). This increase in albumen pH leads to the progressive liquefaction of albumen (decreased viscosity), which is commonly evaluated by measuring Haugh units (HU) (Haugh, 1937). Eggs with high HU values at oviposition and stored for three to four weeks are correlated with higher hatchability compared with eggs exhibiting lower HU values at lay (Hurnik, et al., 1978).

Given the central role of CO_2_ in egg quality degradation, several studies have attempted to maintain an optimal level of CO_2_ within the albumen to mitigate the negative effects of prolonged storage. In some studies, eggs were stored in airtight plastic bags with low gas permeability at different temperatures and storage durations to create a CO_2_-enriched microenvironment around the eggs. These approaches resulted in improved overall hatchability (Becker, et al., 1964, 1968; Gordon and Siegel, 1966) or improved hatchability specifically for storage exceeding seven days (Becker, Spencer and Swartwood, 1964; Kosin and Konishi, 1973; Walsh, et al., 1995). Another experiment showed that storage in bags pre-supplemented with CO_2_ reduced hatchability (Proudfoot, 1964), while a complementary experiment found no significant differences between the various storage conditions (Warren, et al., 1965). Some authors have reported that storage under 4% CO_2_ reduced hatchability, whereas eggs stored for 10 or 21 days under 2% CO_2_ exhibited higher hatchability than eggs stored under ambient air (Sauveur, et al., 1967). Conversely, another article reports reduced hatchability with 1.5% CO_2_ (Kosin and Konishi, 1973), while others observed no effect on hatchability with 0.75% and 1.5% CO_2_ (Reijrink, et al., 2010). Given these contradictory findings, the diversity of experimental factors studied (temperature, CO₂ concentration, storage duration, and genetic line), and the limited control of certain parameters, it remains challenging to determine whether CO₂ exerts a beneficial effect during egg storage.

The objective of our study was to re-evaluate the potential of CO_2_ to preserve egg quality and hatchability following prolonged storage. Unlike most published experiments, eggs were stored in controlled climatic chambers to ensure precise levels of CO_2_, relative humidity, and temperature. Fertilized eggs from a modern layer breed were stored for 6, 13, 20, or 27 days at 12°C, and 80% relative humidity (recommended conditions for prolonged storage), with or without 2% CO_2_. After each storage period under these conditions, eggs were acclimatized for 24 hours at 20°C to mimic hatchery practices. Egg quality was assessed by non-invasive methods (computed tomography scanning and magnetic resonance imaging) to investigate the position of the blastoderm relative to the air cell. Eggs were further weighed and broken to evaluate egg quality using standard measurements: egg mass loss, albumen pH, Haugh units, yolk index and color, and size of the blastoderm. Egg viability and embryo growth under each condition were evaluated after seven days of incubation. This experiment was designed to provide an initial assessment of the effect of CO₂ on egg and blastoderm quality, as well as on early development. It allowed for a preliminary evaluation of the potential benefits and ethical implications of such an approach, prior to potentially implementing this strategy on a larger scale to assess the effect on later stages of development and hatchability.

## Materials and methods

### Egg allocation and storage conditions

The procedure used to store and handle eggs was described previously (Javaloyes, et al., 2026).

Briefly, four hundred eggs from Novobrown laying hens (52 weeks of age) were collected on the day of lay from a local hatchery (Philippe Barat, La Chapelle-sur-Loire, France). Upon arrival at the laboratory, eggs (*n* = 400) were numbered, candled to eliminate cracked eggs, oriented with the air cell upward, and weighed individually (Practum balance, Sartorius, Göttingen, Germany).

In order to ensure homogeneity of egg mass within each experimental batch, a *t*-test was performed to allocate eggs into batches (*n* = 10 eggs per batch) (Blavy, 2025). Half of the batches were stored in a climatic chamber (Memmert ICH 260, Schwabach, Germany) set at 12°C, 80% relative humidity, and 50% ventilation, while the other half were stored in a second climatic chamber (Memmert ICH 260, Schwabach, Germany) at 12°C, 80% relative humidity, 50% ventilation, and 2% CO_2_. Within each climatic chamber, eggs were placed in ascending numerical order, allowing a random spatial distribution of experimental batches in plastic trays, maintaining an empty space between each egg both horizontally and vertically, in order to ensure optimal ventilation within the trays (Javaloyes, et al., 2026). Eggs were stored for 6, 13, 20, or 27 days in climatic chambers, followed by a 24-hour thermal acclimatization at 20°C. This acclimatization was necessary to avoid thermal shock as some eggs were incubated afterwards to evaluate embryo viability. After each storage duration and for each condition (with or without CO_2_), one batch (*n* = 10) was used for egg quality analysis. In parallel, one batch (*n* = 10) was incubated for seven days under standard conditions (55% relative humidity, 37.8°C, automatic turning, Cimuka CT120SH incubator, Ducatillon, France) and then opened for embryonic viability assessment and embryo weighing.

### Egg quality measurements after storage

**Fig. 1:**
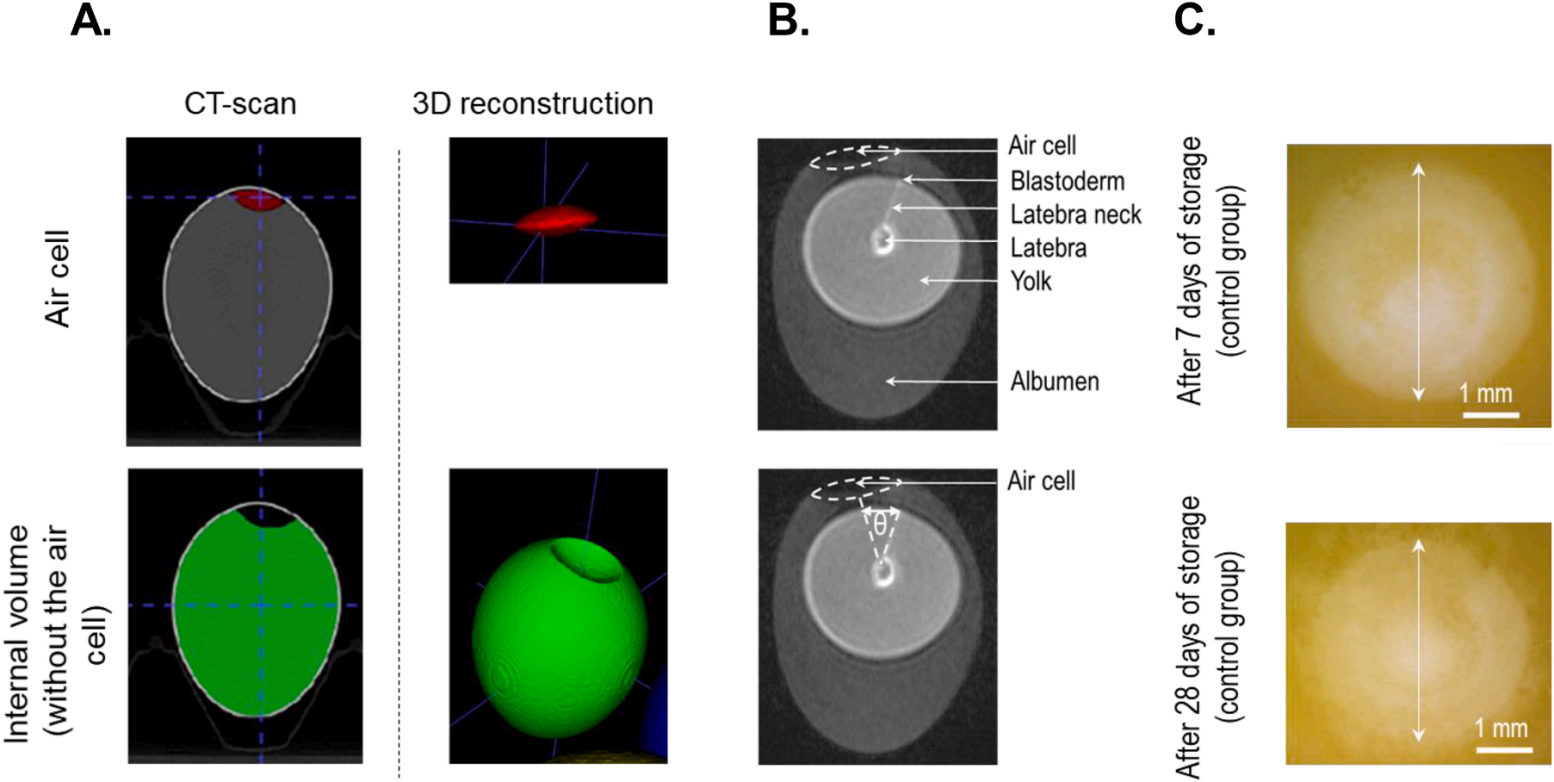
Illustration of imaging techniques. **A**. CT-scan. Using ITK-SNAP tool (Yushkevich and Gerig, 2017), images obtained after CT imaging were analyzed for 3D reconstruction, segmented, and colored for illustration. The air cell and the internal volume of the egg (without the air cell) are illustrated in red and green, respectively. **B.** MRI. Illustration of the method used to locate the blastoderm related to the air cell. **C**. Pictures of the stereomicroscope used to view the blastoderm after 7 and 28 days of storage at 12°C, 80% relative humidity (upper and lower panel, respectively).

On the day following acclimatization, eggs were analyzed using computed tomography (CT) to estimate the percentage of the egg air cell (air cell volume (mm^3^) / total volume of the egg (mm^3^) × 100) (Fig. 1A), and using magnetic-resonance imaging (MRI) to localize the blastoderm on the yolk, and measure its angle relative to the center of the air cell (Fig. 1B).

### CT measurements

Eggs were analyzed by clinical computed tomography (Siemens Somatom® Definition AS 128, Siemens, Germany). The X-ray source was set at 120 kV and 400 mAs. The image acquisition mode was 16 × 0.3 mm with a 512-pixel matrix size with a slice thickness of 0.4 mm and a resolution of 290 µm. Safire U30 and V80 reconstruction filters were used to characterize the internal components of the egg. The Digital Imaging and Communication in Medicine (DICOM) images were converted into Neuroimaging Informatics Technology Initiative (NIfTI) format. The NIfTI images were read with ITK-SNAP software (version 4.2.0-rc.1), to estimate the volume of the air cell and internal components of the eggs (Fig. 1A; Supplementary data S1). The segmentation using “region-growing” option of the software was done manually.

### MRI analyses

MRI analyses were performed using Syngo.Via software (VA70) on a 3T MRI scanner (Siemens Magnetom® Verio, Erlangen, Germany). Three-dimensional T1-weighted images were acquired using an MPRAGE (magnetization prepared rapid gradient echo) sequence. Acquisition parameters were as follows: TR = 2,500 ms; TE = 3.21 ms; TI = 900 ms; flip angle = 12 °; matrix = 192 × 192; slice thickness = 0.5 mm, resulting in an isotropic voxel size of 0.5 × 0.5 × 0.5 mm³. A bandwidth of 150 Hz/pixel and one excitation (NEX = 1) were used. The position of the blastoderm on the MRI images was estimated based on T_1_ images of the yolk and the albumen. This imaging technique clearly allows the distinction of white yolk and yellow yolk, thereby enabling the localization of the neck of the latebra (Fig. 1B; Supplementary data S1).

### Physicochemical measurements

After imaging analysis, eggs were weighed (Practum balance, Sartorius, Göttingen, Germany) to calculate mass loss after storage related to egg dehydration ((egg weight at lay – egg post-storage weight) / egg weight at lay × 100). Eggs were then analyzed using a DET6000 device (DET6000, Nabel, Kyoto, Japan) to assess various quality parameters, including breaking strength, HU, yolk color, yolk index, and shell thickness.

The yolk and albumen were subsequently separated to measure albumen pH (GLP Sension+ Ph31, Hach, Lognes, France). The blastoderm located on the yolk was observed using a stereomicroscope (× 4 objective, Mantis Elite, Vision Engineering, Le Plessis-Pâté, France) connected to a computer for measurement of its diameter (ImageJ, v1.53e) (Fig. 1C). Finally, eggshells from each egg were rinsed, dried for 2 hours at 110°C in an oven (AC120, FIRLABO SA, Meyzieux, France), and then weighed to estimate the individual percentage of total eggshell mass relative to egg mass (Practum balance, Sartorius, Göttingen, Germany).

### Incubation procedure

After each storage period, batches from each condition were incubated in an automatic incubator (Cimuka 120SH, Ducatillon, Fretin, France) set at 37.8°C, 55% relative humidity, with automatic egg turning. On day 7 of incubation, eggs were opened individually, alternating between eggs from the control and 2% CO₂ storage groups. Eggs were broken, and the contents of the eggs were poured in a Petri dish. The yolk sac and the chorioallantoic membrane were separated carefully from the embryo with surgical scissors. Each embryo was dried on absorbent paper to remove excess liquid, quickly decapitated, and weighed (Sartorius Practum, 124-1S Analytical Balance, 120 *g* x 0.0001 *g*, Göttingen, Germany). Embryos were considered viable when cardiac activity was detected at dissection. Embryonic mortality was recorded only in fertilized eggs showing either a small dead embryo or the presence of a vascularized yolk sac.

### Statistical analysis

As mentioned above, the allocation of eggs into experimental batches was performed using a *t*-test based on egg mass (Blavy, 2025). This procedure ensured a homogeneous distribution of eggs according to their mass between batches, before storage. This distribution was re-assessed using a Kruskal-Wallis test (*p* < 0.05) after removal of cracked eggs or eggs presenting biological abnormalities, in order to verify batch balance (RStudio, version R-4.4.2) and avoid any bias related to differences in egg mass between batches.

Given the small sample sizes and the non-normality observed for most parameters, homogeneous non-parametric approaches were used for all analyses. Prior to statistical analysis, outliers were removed using the ROUT method set at 1% (Motulsky and Brown, 2006). Five values were removed (one value for egg mass loss, one value for yolk color, one value for air cell volume, and two values for latebra neck angle). A principal component analysis (PCA) was performed to explore relationships between parameters and calculate correlation factors (Spearman non-parametric correlations, two-tailed). Since some eggs lacked values for certain parameters (due to outliers and missing data), PCA was performed on 66 of the 76 eggs analyzed.

Data related to eggshell quality, egg quality, and embryo relative mass were analyzed using the Aligned Rank Transform (ART) ANOVA, a non-parametric approach used to test main effects and interactions (Leys and Schumann, 2010). This approach was used because several variables did not meet the criteria for normality (Shapiro-Wilk test, *p* < 0.05) or homoscedasticity (Levene test, *p* < 0.05), and the number of replicates per group was small (≤ 10).

Storage duration, storage condition, and their interaction were included as fixed effects. When a significant effect of storage duration or a significant interaction was detected, pairwise comparisons were performed using the “art.con()” function from the ARTool package with Bonferroni adjustment for multiple testing. Statistical analyses were conducted using RStudio (version R-4.4.2).

Embryonic viability was analyzed using Fisher’s exact test (GraphPad Prism version 10.6.1 for Windows, Boston, Massachusetts USA, www.graphpad.com).

## Results

### Principal component analysis (PCA) and correlations between egg quality parameters

**Fig. 2:**
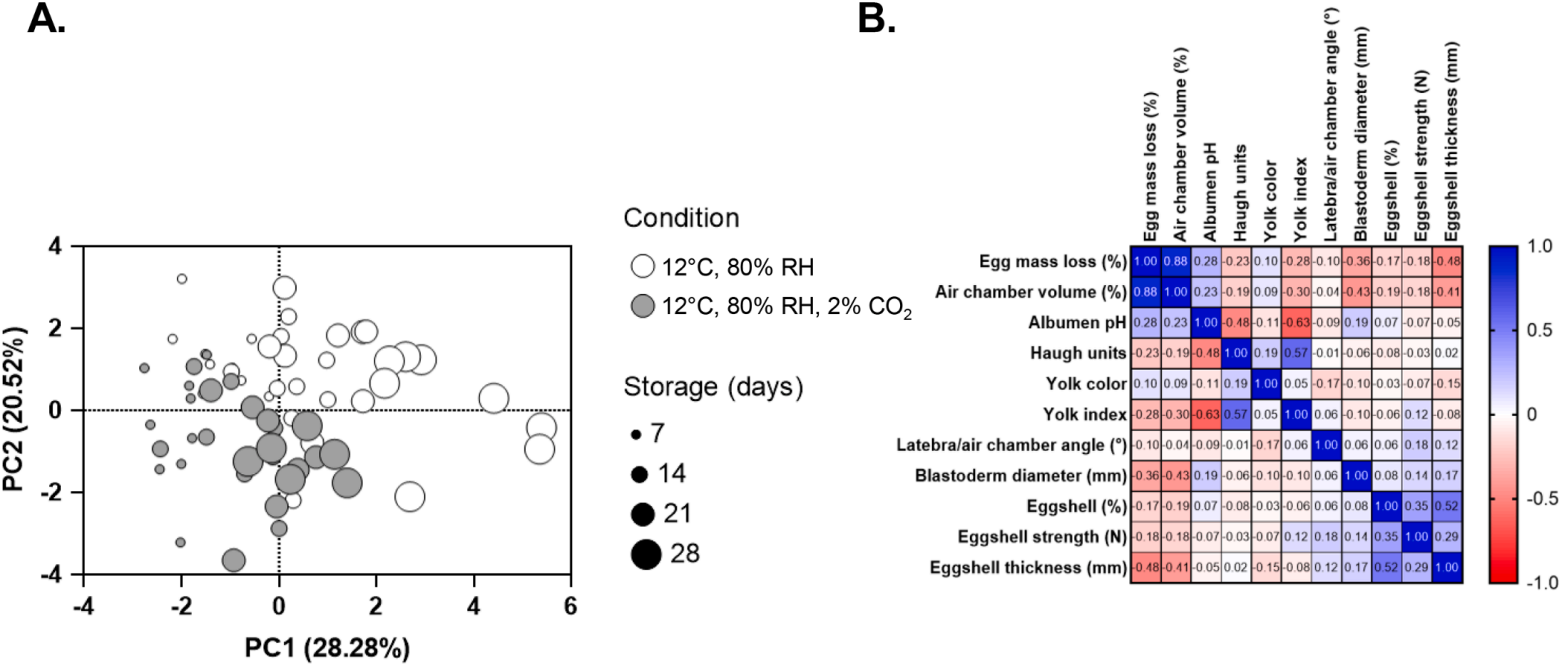
Principal component analysis (PCA) and Spearman correlations between parameters. **A.** PCA. PCA was performed on 66 eggs out of the 76 eggs analyzed (see Material and Methods). The PC1 and PC2 explained 48.8% of the total variance. The five main variables contributing to PC1 are egg mass loss (0.266), air cell volume (0.203), yolk index (0.129), albumen pH (0.105), and Haugh units (0.089). The five main variables contributing to PC2 are eggshell percentage (0.162), albumen pH (0.153), Haugh units (0.133), yolk index (0.128), and eggshell thickness pH (0.118). **B.** Spearman correlations. Positive correlations are illustrated in blue while negative ones are shown in red. The higher the absolute value, the stronger the correlation (negative or positive).

PCA performed on all eggs (control batches, CO_2_ batches, after 7, 14, 21, or 28 days of storage) revealed a clear separation between eggs stored with CO_2_ and those stored without CO_2_ and showed that eggs stored under CO_2_ were more clustered compared with control eggs (Fig. 2A). Regardless of the atmosphere (with or without CO_2_), eggs stored for a longer period (21 and 28 days) tended to cluster on the right side of the graph (Fig. 2A).

A very strong positive correlation was observed between egg mass loss and air cell volume (*r* = 0.88; *p* < 0.0001) (Fig. 2B). These two parameters were positively correlated with albumen pH with *r* = 0.28 (*p* < 0.05) and 0.23 (*p* < 0.05), respectively. In contrast, they were negatively correlated with yolk index (*r* = - 0.28, *p* < 0.05; *r* = −0.30, *p* < 0.01, respectively), blastoderm diameter (*r* = −0.36, *p* < 0.005; *r* = −0.43; *p* < 0.0005, respectively), and eggshell thickness (*r* = −0.48, *p* < 0.0001; *r* = −0.41, *p* < 0.0005, respectively) (Fig. 2B). A strong negative correlation was also highlighted between yolk index and albumen pH (*r* = −0.63, *p* < 0.0001), and a positive correlation between yolk index and Haugh units (*r* = 0.57, *p* < 0.0001). As expected, Haugh units were found to be negatively correlated with albumen pH (*r* = −0.48, *p* < 0.0001) and eggshell strength was positively correlated with eggshell thickness (*r* = 0.29, *p* < 0.05) and eggshell percentage (*r* = 0.35, *p* < 0.05). In addition, eggshell thickness and eggshell percentage were positively correlated (*r* = 0.52, *p* < 0.001). All other correlations were not statistically significant (*p* > 0.05).

### Eggshell quality after storage

**Fig. 3:**
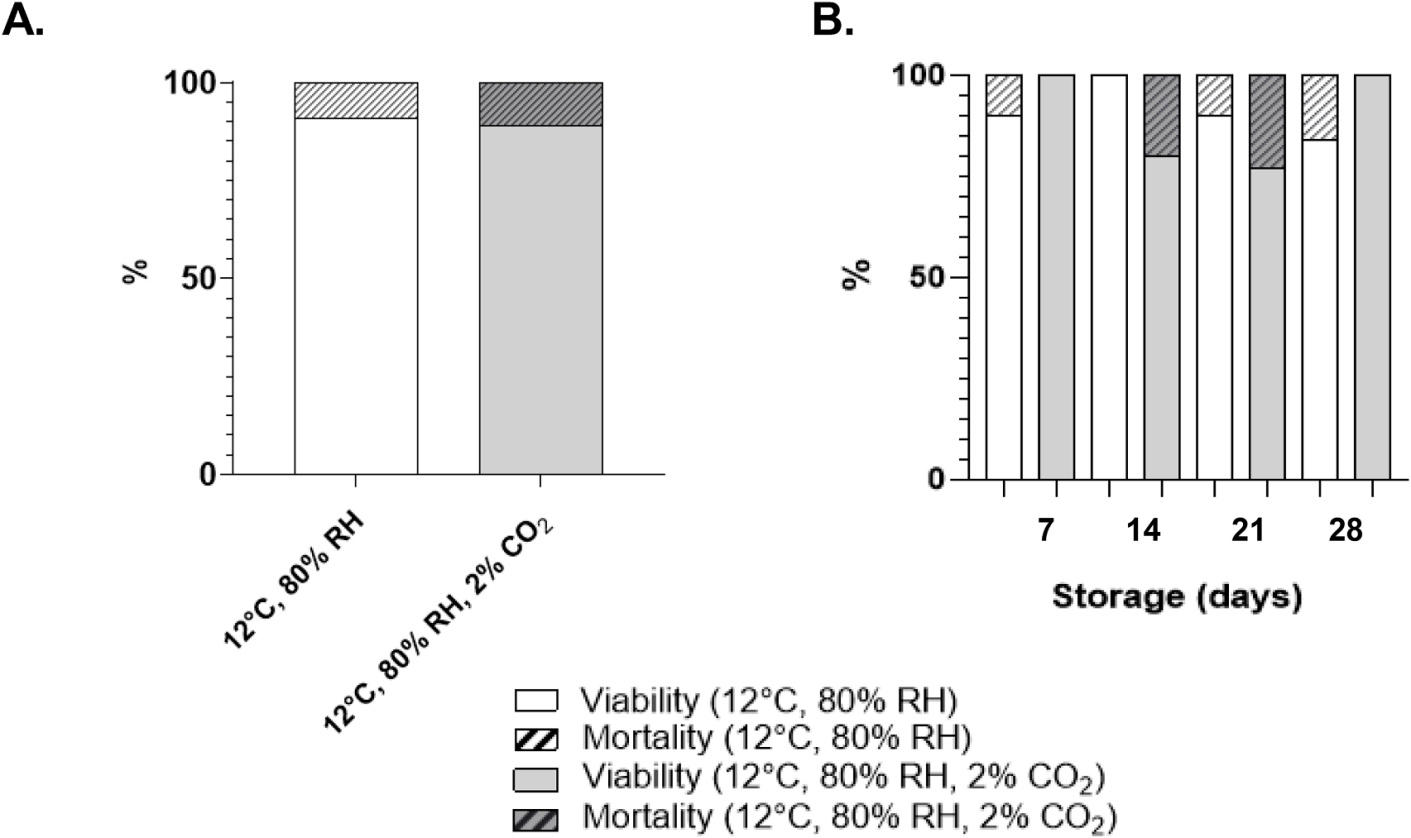
Viability of seven-day-old embryos (ED7) following storage at 12°C, 80% relative humidity with or without 2% CO_2_, and for 7, 14, 21, and 28 days. **A**. Viability according to condition of storage (all storage duration combined). **B**. Viability according to condition and duration of storage. Data are expressed as percentage of total eggs per batch (*n* ≈ 16 per condition).

Verifying the quality of the eggshell is crucial in this type of experiment, as a defective eggshell has a direct impact on the egg’s internal structures resulting in increased water loss and deterioration of internal components. The 400 eggs received were carefully inspected by candling to remove any cracked eggs prior to their distribution into batches. To avoid any additional bias in the data analysis when comparing batches, we checked eggshell integrity and quality by measuring breaking strength, shell thickness, and shell mass percentage (Table 1, Fig. 3).

**Table 1:** Eggshell quality after storage for 7, 14, 21, and 28 days with or without 2% CO2 at 12°C, 80% relative humidity (*n* ≈ 10 per batch). For more details about the measurements, refer to Material and methods.

	Eggshell breaking strength (N)	Eggshell thickness (mm)	Eggshell (%)
Condition			
Control	43.19 ± 6.95	0.44 ± 0.06	10.28 ± 0.75
CO_2_ (2%)	43.27 ± 5.58	0.43 ± 0.04	10.15 ± 0.47
Storage (days)			
7	45.18 ± 4.48	0.46 ± 0.04 ^a^	10.48 ± 0.52
14	41.37 ± 5.09	0.43 ± 0.05 ^ab^	10.02 ± 0.63
21	44.19 ± 7.32	0.44 ± 0.06 ^a^	10.38 ± 0.46
28	42.38 ± 7.75	0.40 ± 0.02 ^b^	10.01 ± 0.83
Storage × Condition			
7 × Control	45.19 ± 5.44	0.46 ± 0.04	10.66 ± 0.43
14 × Control	41.55 ± 3.52	0.43 ± 0.06	10.13 ± 0.62
21 × Control	46.59 ± 7.25	0.46 ± 0.07	10.49 ± 0.50
28 × Control	40.12 ± 9.37	0.40 ± 0.03	9.86 ± 1.07
7 × CO_2_ (2%)	45.17 ± 3.45	0.45 ± 0.03	10.28 ± 0.56
14 × CO_2_ (2%)	41.19 ± 6.50	0.43 ± 0.04	9.91 ± 0.66
21 × CO_2_ (2%)	41.80 ± 7.01	0.42 ± 0.04	10.27 ± 0.42
28 × CO_2_ (2%)	45.21 ± 4.06	0.40 ± 0.02	10.17 ± 0.46
*p*-values			
Condition	0.8743	0.4607	0.4994
Storage	0.1713	**0.0003**	**0.0322**
Storage × Condition	0.0681	0.2916	0.4040

^a-b^ Means having different letters in each column are significantly different (ART ANOVA, post-hoc test; *p* < 0.05). For the main storage and condition effects and the condition x storage interaction, significant values (ART ANOVA; *p* < 0.05) are highlighted in bold.

The ART ANOVA statistical analysis did not reveal any significant differences in the breaking strength (Table 1). In contrast, significant differences in shell thickness and eggshell proportion were observed between control and CO_2_ batches (*p* = 0.0003 and *p* = 0.0322, respectively). However, post-hoc comparisons following ART ANOVA did not reveal any significant pairwise differences in eggshell proportion, while a significant difference in the eggshell thickness parameter was observed between conditions when comparing eggs stored for 7 and 21 days with those stored for 28 days (*p* = 0.0001 and *p* = 0.0421, respectively; Table 1).

### Internal egg quality after storage

Internal egg quality was assessed using non-invasive techniques (egg mass loss after storage, air cell volume, and blastoderm location) and invasive techniques (albumen pH, HU, yolk index, yolk color).

Concerning the two parameters related to egg dehydration (egg mass loss and the volume of the air cell), statistical analyses revealed a storage × condition interaction (Table 2). These two parameters followed a similar trend in both experimental conditions with an increase in egg mass loss as early as 14 days of storage compared with 7 days of storage, but the mean values of these parameters for eggs stored with or without CO_2_ were not significantly different.

**Table 2:** Egg mass loss and albumen quality after storage for 7, 14, 21, and 28 days with or without 2% CO_2_ at 12°C, 80% relative humidity (*n* ≈ 10 per batch). For more details about the measurements, refer to Material and methods.

	Egg mass loss (%)	Air cell (%)	Albumen pH	Haugh Units
Condition				
Control	1.26 ± 0.57	1.70 ± 0.60	9.21 ± 0.10	78.47 ± 8.65 ^b^
CO_2_ (2%)	1.09 ± 0.39	1.51 ± 0.47	8.82 ± 0.12	85.49 ± 6.60 ^a^
Storage (days)				
7	0.64 ± 0.07	1.03 ± 0.12	9.02 ± 0.13	85.44 ± 6.34
14	1.00 ± 0.14	1.44 ± 0.16	8.96 ± 0.22	83.45 ± 8.55
21	1.27 ± 0.24	1.73 ± 0.32	9.01 ± 0.25	78.16 ± 9.25
28	1.84 ± 0.39	2.30 ± 0.45	9.09 ± 0.25	80.61 ± 8.08
Storage × Condition				
7 × Control	0.67 ± 0.08 ^f^	1.08 ± 0.15 ^d^	9.12 ± 0.08 ^c^	82.50 ± 5.18
14 × Control	1.03 ± 0.10 ^de^	1.46 ± 0.10 ^c^	9.17 ± 0.05 ^bc^	81.08 ± 9.43
21 × Control	1.33 ± 0.17 ^bc^	1.81 ± 0.41 ^b^	9.22 ± 0.07 ^b^	73.19 ± 9.48
28 × Control	2.13 ± 0.33 ^a^	2.47 ± 0.38 ^a^	9.32 ± 0.03 ^a^	76.99 ± 7.80
7 × CO_2_ (2%)	0.61 ± 0.04 ^f^	0.97 ± 0.05 ^d^	8.91 ± 0.07 ^d^	88.38 ± 6.25
14 × CO_2_ (2%)	0.98 ± 0.18 ^e^	1.42 ± 0.20 ^c^	8.76 ± 0.10 ^e^	85.82 ± 7.27
21 × CO_2_ (2%)	1.21 ± 0.29 ^cd^	1.64 ± 0.19 ^bc^	8.79 ± 0.16 ^e^	83.12 ± 6.03
28 × CO_2_ (2%)	1.56 ± 0.18 ^ab^	2.09 ± 0.46 ^ab^	8.84 ± 0.08 ^de^	84.62 ± 6.65
*p*-values				
Condition	**< 0.0001**	**< 0.0001**	**< 0.0001**	**< 0.0002**
Storage	**< 0.0001**	**< 0.0001**	**< 0.0001**	**0.0352**
Storage × Condition	**0.0003**	**0.0474**	**< 0.0001**	0.7955

^a-e^ Means having different letters in each column are significantly different (ART ANOVA, post-hoc test; *p* < 0.05). For the main storage and condition effects and the condition x storage interaction, significant values (ART ANOVA; *p* < 0.05) are highlighted in bold.

Albumen quality was assessed by measuring albumen pH and HU. A storage × condition interaction was found for albumen pH (Table 2). Over the storage period, the pH values of control batches were significantly higher than those of CO_2_ batches. In addition, control batches exhibited a gradual increase in albumen pH over time. In contrast, the pH of albumen in CO_2_-batches decreased from day 7 to day 14 of storage then remained stable until 28 days of storage.

No storage × condition interaction was found for Haugh units. However, the mean value of Haugh units in CO_2_ batches was significantly higher than that of control batches (*p* < 0.0002, Table 2). In contrast, for the storage main effect (*p* = 0.0352), the pairwise comparison did not reveal any significant difference in Haugh units between batches over the storage period (Table 2).

The yolk quality was assessed by measuring yolk color and index. Concerning yolk color, a main effect of condition was found, with control eggs having a slightly lighter yolk color value compared with CO_2_ eggs (*p* = 0.0338, Table 3). As for yolk index, a storage × condition interaction was found. The values of yolk index from CO_2_-batches remained stable over the storage period, while those from control batches gradually decreased over time. After 21 and 28 days of storage, the yolk index of control batches was significantly lower than that of eggs stored for 7 days and that from CO_2_-egg stored for 21 and 28 days (Table 3).

**Table 3:** Characteristics of the egg yolk and blastoderm after storage for 7, 14, 21, and 28 days with or without 2% CO_2_ at 12°C, 80% relative humidity (*n* ≈ 10 per batch). For more details about the measurements, refer to Material and methods.

	Yolk color	Yolk index	Latebra neck angle (°)	Blastoderm diameter (mm)
Condition				
Control	5.06 ± 0.42 ^b^	0.40 ± 0.03	18.58 ± 11.59	3.48 ± 0.57 ^a^
CO_2_ (2%)	5.29 ± 0.50 ^a^	0.45 ± 0.03	20.59 ± 13.55	3.21± 0.48 ^b^
Storage (days)				
7	5.20 ± 0.58	0.44 ± 0.04	19.80 ± 14.19	3.75 ± 0.46 ^a^
14	5.02 ± 0.47	0.42 ± 0.03	21.95 ± 13.69	3.37 ± 0.45 ^abc^
21	5.21 ± 0.45	0.42 ± 0.04	20.12 ± 12.65	3.25 ± 0.54 ^bc^
28	5.28 ± 0.36	0.41 ± 0.05	16.11 ± 9.04	3.04 ± 0.50 ^c^
Storage × Condition				
7 × Control	5.00 ± 0.49	0.41 ± 0.02 ^abc^	16.93 ± 8.56	3.91 ± 0.43
14 × Control	4.90 ± 0.55	0.41 ± 0.02 ^bc^	20.74 ± 13.12	3.55 ± 0.49
21 × Control	5.18 ± 0.30	0.39 ± 0.03 ^cd^	21.88 ± 15.63	3.50 ± 0.43
28 × Control	5.18 ± 0.26	0.37 ± 0.04 ^d^	14.70 ± 7.91	3.00 ± 0.58
7 × CO_2_ (2%)	5.40 ± 0.62	0.46 ± 0.04 ^a^	22.98 ± 18.68	3.59 ± 0.44
14 × CO_2_ (2%)	5.13 ± 0.38	0.43 ± 0.03 ^abc^	23.17 ± 14.85	3.20 ± 0.35
21 × CO_2_ (2%)	5.23 ± 0.58	0.45 ± 0.03 ^ab^	18.14 ± 8.83	2.99 ± 0.53
28 × CO_2_ (2%)	5.41 ± 0.43	0.45 ± 0.02 ^ab^	17.52 ± 10.33	3.08 ± 0.44
*p*-values				
Condition	0.**0338**	**< 0.0001**	0.7727	**0.0081**
Storage	0.1649	**0.0271**	0.6348	**0.0002**
Storage × Condition	0.6651	**0.0496**	0.9029	0.2415

^a-e^ Means having different letters in each column are significantly different (ART ANOVA, post-hoc test; *p* < 0.05). For the main storage and condition effects and the condition x storage interaction, significant values (ART ANOVA; *p* < 0.05) are highlighted in bold.

Concerning blastoderm positioning, MRI analysis did not reveal any significant differences in the orientation of the neck of latebra —and, consequently, of the blastoderm— relative to the air cell (Table 3). In contrast, the analysis of the blastoderm size using stereomicroscopic imaging revealed main effects of storage and condition (*p* = 0.0002 and *p* = 0.0081, respectively) but no interaction between the two factors (Table 3). The diameter of the blastoderm in control eggs was significantly higher than that of CO_2_ eggs, while eggs stored for 21 and 28 days exhibited smaller blastoderms than those stored for 7 days (Table 3).

### Embryonic viability and embryo mass

After 7 days of incubation, eggs were opened, the viability of embryos was assessed by counting the number of live and dead embryos per batch and expressing viability as a percentage of total eggs (Fig. 3A and 3B). The results showed no significant differences in viability among the experimental conditions (duration, with or without 2% CO_2;_ Fig. 3A and 3B). Therefore, no effect of storage duration and/or storage condition on the embryonic viability was observed in our experimental conditions.

No storage × condition interaction was found for the embryo mass (expressed as percentage relative to egg mass prior to incubation). However, embryos from CO_2_ eggs were smaller than those from control eggs (*p* < 0.0001), and results showed that the embryo mass decreased progressively with storage duration (*p* < 0.0001) (Table 4). Interestingly a significant and high negative correlation of embryo relative mass was found with dehydration parameters (egg mass loss, *r* = −0.49, *p* < 0.0001); air cell volume, *r* = −0.44, *p* < 0.0005) and a positive correlation of this parameter with blastoderm diameter (*r* = 0.42, *p* < 0.0005) (Supplementary Data 2). In addition, a significant negative correlation was identified between embryo mass at ED7 and yolk color (*r* = −0.24, *p* < 0.05).

**Table 4:** Relative embryo mass after incubation for seven days (ED7), following storage for 7, 14, 21, and 28 days with or without 2% CO_2_ at 12°C, 80% relative humidity (*n* ≈ 10 per batch). For more details about the measurements, refer to Material and methods.

	ED7-EMBRYO (%)
Condition	
Control	0.95 ± 0.25 ^a^
CO_2_ (2%)	0.78 ± 0.27 ^b^
Storage (days)	
7	1.00 ± 0.20 ^ab^
14	0.99 ± 0.20 ^a^
21	0.86 ± 0.18 ^b^
28	0.54 ± 0.19 ^c^
Storage × Condition	
7 × Control	1.00 ± 0.14
14 × Control	1.09 ± 0.09
21 × Control	0.98 ± 0.18
28 × Control	0.67 ± 0.12
7 × CO_2_ (2%)	0.99 ± 0.25
14 × CO_2_ (2%)	0.88 ± 0.23
21 × CO_2_ (2%)	0.79 ± 0.14
28 × CO_2_ (2%)	0.43 ± 0.18
*p*-values	
Condition	**< 0.0001**
Storage	**< 0.0001**
Storage × Condition	0.4632

^a-d^ Means having different letters in each column are significantly different (ART ANOVA, post-hoc test; *p* < 0.05). For the main storage and condition effects and the condition x storage interaction, significant values (ART ANOVA; *p* < 0.05) are highlighted in bold.

## Discussion

Egg internal quality is intimately linked to the diffusion of gases (water and CO_2_) through eggshell pores. Shell conductance is an inherent property influenced by shell thickness, pores number, egg mass, and hen age (Onagbesan, et al., 2007). Previous work proposed a model of CO_2_ diffusion as a function of storage duration and temperature, in which the gas is considered denser than air, and follows a four-step process: (1) adsorption of CO_2_ at the shell surface, (2) gravity-driven downward diffusion, (3) mixing of diffusion layers, and (4) formation of a CO_2_ flux toward the ground. Comparisons of diffusion profiles at 4°C, 25°C, and 35°C confirmed that lower storage temperatures reduce both the amount and the speed of CO_2_ released from eggs (Wang, et al., 2022). In addition to temperature, eggshell conductance also influences gas diffusion. Shell conductance is affected by several factors, including the age of the laying hen, egg size, and shell pore density (Ar, et al., 1974). Cracked or fractured shells allow greater gas diffusion. In our study, we did not directly calculate conductance per egg, but we verified the integrity and uniformity of shell quality across experimental groups by assessing shell strength, measuring shell thickness and shell mass proportion. Moreover, given that egg mass can also influence gas diffusion, we worked with batches that had a similar mean egg mass (*t*-test, (Blavy, 2025)). No significant differences were observed in shell strength or proportion. In contrast, eggshell thickness was slightly lower in eggs stored for 28 days compared with those stored for 7 or 21 days, but it did not differ from that of eggs stored for 14 days (Table 1). Due to the high sensitivity of the measurement, this observation requires further confirmation. Overall, eggshell quality was comparable across all eggs used for quality analyses, which was very important to avoid any bias of interpretation due to broken eggs or fragile shells, both of which are associated with increased gas diffusion. Interestingly enough, the shell thickness was inversely correlated with dehydration parameters (*r* = −0.48 for mass loss, *r* = −0.41 for air cell volume), confirming that thicker eggshells limit water loss during storage. As expected, the eggshell thickness was also positively correlated with the eggshell percentage (*r* = 0.52) and, to a lesser extent, shell strength (*r* = 0.29), confirming their interrelated role in the structural integrity of the eggshell.

Egg dehydration is a well-known process that occurs continuously via gas exchange, primarily from the albumen. The extent of egg dehydration strongly depends on storage conditions. Intact eggs stored at 10°C and 80% relative humidity lose approximately 0.015 g/day (Romanoff and Romanoff, 1949). In our study, the mean egg mass over the storage period was 0.048 ± 0.01 g/day (*n* ≈ 40) and 0.042 ± 0.014 g/day (*n* ≈ 40) for the control and CO_2_ groups, respectively, which is slightly higher than the value previously published. This discrepancy may be due to the temperature (10°C in (Romanoff and Romanoff, 1949) *versus* 12°C in the present study), and to the delay between hatching time and the time when eggs were stored. In our study, eggs were collected the day of laying from the farm, and we showed previously that eggs lose about 0.2% of their initial weight after three days of storage at 12°C, 80% relative humidity (Javaloyes, et al., 2026). In addition, it has to be highlighted that those eggs were acclimatized for 24 hours at 20°C, as commonly performed in commercial hatcheries, to avoid thermal shock, especially after storage at cool temperatures. This processing step is associated with an increase in egg weight loss compared with eggs that are not acclimatized (Javaloyes, et al., 2026). Concomitantly to egg mass loss, the air cell volume assessed by CT scanning showed a gradual increase during storage, which is in accordance with the strong correlation observed between these two parameters (*r* = 0.88, Fig. 2B).

Our results confirm that prolonged storage at 12°C, 80% relative humidity is associated with a small increase in dehydration but, interestingly, storing egg with 2% CO_2_ partly mitigates the effect (storage × condition interaction).

Albumen alkalinization during storage is well documented. Freshly laid eggs have a pH of approximately 7.6 at oviposition, which gradually increases due to CO_2_ loss and the bicarbonate–CO_2_ buffering mechanism (Romanoff and Romanoff, 1949). The rate of pH increase depends on CO_2_ diffusion, which is itself influenced by storage temperature (Sharp and Powell, 1931; Wang, Cao, Wang and Ma, 2022). External CO_2_ partial pressure also affects albumen pH: in 100% CO_2_, pH can decrease to 6.5 (Brooks and Pace, 1938). Another experiment showed that storage at 10°C under 2% CO_2_ maintained albumen pH over time (Sauveur, Ferré and Lacassagne, 1967). Our results confirm these findings, as albumen pH values remained stable from day 14 to day 28 in CO_2_ eggs, whereas they gradually increased over time in control eggs (storage × condition interaction, *p* < 0.001). The higher albumen pH observed in our study compared with previously reported values (8.79 vs. 7.77, (Sauveur, Ferré and Lacassagne, 1967)) is likely due to the 24-hour acclimatization period at 20°C prior to analysis. Indeed, our previous work indicated that a 24-hour acclimatization following six days of storage at 12°C, 80% relative humidity, and 2% CO_2_ resulted in a 0.7-unit increase in albumen pH (Javaloyes, et al., 2026). As expected, albumen pH was found to be inversely correlated with Haugh units (*r* = −0.48). In addition, Haugh units in CO_2_ batches were significantly higher than those in control batches (*p* < 0.0002), but unlike albumen pH, no interaction with storage duration was observed. In both CO_2_ and control batches, eggs exhibited very high HU values (≥ 72), corresponding high-grade eggs (Table 2).

Similar to HU, yolk index was negatively correlated with albumen pH (*r* = −0.63). It is assumed that the alkalinization of the albumen surrounding the yolk may affect the vitelline membrane, by altering its protein structure and reducing membrane integrity (Hervé et al., 2026), thereby leading to lower yolk index values. Our data showed that yolk index decreased overtime in control batches but remained stable in CO_2_ batches, and was higher than that of control eggs stored for 21 and 28 days (storage × condition interaction; *p* < 0.0496). Yolk color was affected only by the main effect of condition, with values slightly higher in CO_2_ batches compared with control batches (*p* = 0.0338). Previous studies have highlighted that storage has little effect on this parameter, because yolk color depends primarily on the hen’s diet (Romanoff and Romanoff, 1949). We hypothesize that the presence of CO_2_ combined with the high viscosity of albumen may slightly alter the apparent color at the yolk surface (increased of approximately 0.23 units). Indeed, albumen enriched in CO_2_ is highly viscous (as reflected by the higher HU values observed in CO_2_ batches compared with control eggs, Table 2) and slightly opaque.

Blastoderm position in the egg was measured by MRI, using the central air cell axis and the neck of the latebra. The latebra is a spherical structure located at the center of the yolk and composed of white yolk, while the neck of the latebra extends toward the nucleus of Pander, which is itself an extension of the inner surface of the blastoderm (Réhault-Godbert and Guyot., 2026; Romanoff and Romanoff, 1949). The latebra and the latebra neck are readily visualized by MRI (Fig. 1B). The blastoderm is located at the end of the neck of the latebra, whereas the air cell and its volume can be visualized by CT scanning (Fig. 1A). The rationale for including this measurement in our protocol was based on the hypothesis that the progressive rotation of the blastoderm toward the air cell could promote its dehydration and consequently increase its deterioration. Limiting this mechanism by maintaining high albumen viscosity via 2% CO_2_ in the storage atmosphere could therefore protect the blastoderm from degradation. In our experiment, blastoderm position at the yolk surface was not affected by either storage duration or CO_2_ treatment. Our data suggest that the absence of changes in blastoderm angle relative to the air cell may be due to the high albumen viscosity (HU > 72) observed across all batches. Indeed, published studies have shown that when eggs are stored large-end up, blastoderm positioning is not solely determined by yolk density but is strongly influenced by albumen properties (Besch and Sluka, 1966). Notably, after 96 hours of incubation, blastoderm deviation decreased (approximately 20° versus approximately 27° before incubation), indicating that albumen physicochemical properties directly influence blastoderm positioning. Although, under our experimental conditions, we could not correlate blastoderm position with potential deterioration of its cytoarchitecture, we believe that this non-invasive approach is highly promising for studying changes in the yolk and the blastoderm without opening the egg. In contrast, we observed a significant decrease in blastoderm diameter from 21 days of storage onwards (Table 3; storage main effect; *p* = 0.0002), while CO_2_ eggs displayed smaller blastoderms than control eggs (*p* = 0.0081). The reduction in blastoderm diameter following prolonged storage has been reported previously (Arora and Kosin, 1966; Pokhrel et al., 2018) and likely results from cell apoptosis (Brady et al., 2022). Similarly, it would be interesting to further investigate the effects of CO_2_ on morphology, and metabolic activities of blastoderm cells, as described previously (Pokhrel, et al., 2018).

Following seven days of incubation (ED7), no differences in embryonic viability were observed among storage conditions or storage duration (Fig. 3A and 3B). However, these findings should be interpreted with caution given the limited number of replicates used in the present study. Statistical analyses revealed that mean embryo mass (expressed relative to egg mass) at ED7 was significantly lower in the CO_2_ group than in the control group (condition main effect, *p* < 0.0001, Table 4) and decreased over time (storage main effect, *p* < 0.0001; Table 4). These results suggest that prolonged storage and/or CO₂ treatment may adversely affect embryonic growth and development, potentially as a consequence of differences in egg quality prior to incubation, a delayed resumption of embryonic development following incubation, or a combination of both factors. Spearman correlation revealed a positive correlation between embryo relative mass and blastoderm diameter (Supplementary data S2), which is consistent with the hypothesis that a smaller blastoderm diameter may result from apoptosis, thereby impair early development of embryos (fewer initial cells and a greater delay in development resumption (Brady et al., 2022)). In addition, negative correlations were observed between embryo relative mass and dehydration parameters (egg mass loss and air cell volume; supplementary data S2). Although our viability results provide no evidence that these changes impair survival, blastoderms from eggs stored for prolonged periods under non-optimal conditions (temperature >12°C, relative humidity <80%) may be more susceptible to dehydration and cell death, potentially compromising developmental resumption upon incubation.

Collectively, these data demonstrate that eggs stored under 2% CO₂ at 12°C and 80% relative humidity exhibit improved egg quality parameters (Haugh units, albumen pH, and yolk index) after prolonged storage compared with control eggs stored without CO₂. However, CO₂ did not prevent dehydration over time, although it slightly mitigated its effects. None of these positive changes appeared to significantly influence embryonic viability or developmental resumption. Nevertheless, embryos from CO₂-treated eggs were significantly smaller than those from control eggs, which may be partly explained by the smaller blastoderm diameter observed before incubation.

Based on these findings, it would be worthwhile to evaluate lower CO₂ concentrations using a larger number of replicates and to extend incubation to 10 days rather than 7 days in order to better characterize physiological differences in embryonic development between treatments over time. Finally, although no differences in blastoderm positioning were detected among treatments, MRI and CT imaging proved to be valuable non-invasive tools for quantifying internal egg parameters and monitoring changes in internal structures such as air cell volume, and yolk and blastoderm positions.

## Ethics Statement

The procedure used to euthanize embryos (decapitation) is in compliance with the European legislation on the “Protection of Animals Used for Experimental and Other Scientific Purposes” (2010/63/UE), and follows the guidelines approved by the institutional animal care and use committee (IACUC). The study complies the ARRIVE 2.0 guidelines (Percie du Sert, et al., 2020).

## CRediT authorship contribution statement

**P. Javaloyes:** Writing – review & editing, Writing – original draft, Visualization, Validation, Methodology, Investigation. **N. Bernardet:** Writing – review & editing, Formal analysis. **F. Elleboudt:** Writing – review & editing, Investigation, Formal analysis. **H. Adriaensen:** Writing – review & editing, Investigation, Formal analysis. **A. Collin:** Writing – review & editing, Supervision, Formal analysis. **S. Réhault-Godbert:** Writing – review & editing, Writing – original draft, Visualization, Validation, Supervision, Project administration, Methodology, Investigation, Funding acquisition, Formal analysis, Conceptualization.

## Disclosures

The authors declare no conflict of interest. The funders had no role in the design of the study; in the collection, analyses, or interpretation of data; in the writing of the manuscript, or in the decision to publish the results.

## Appendix Supplementary materials

Image, application 1

Image, application 2

## Footnotes

Supplementary material associated with this article can be found, in the online version, at doi:10.1016/j.psj.2026.107466.
