# Supplementary material for: Impact of 2% carbon dioxide during storage on egg quality and early embryo development
Source: Poult Sci. 2026 Jul 17;105(11):107466. doi: 10.1016/j.psj.2026.107466 (PMC13453520; doi:10.1016/j.psj.2026.107466)
Supplement: Supplementary file 1 [file mmc1.pdf]

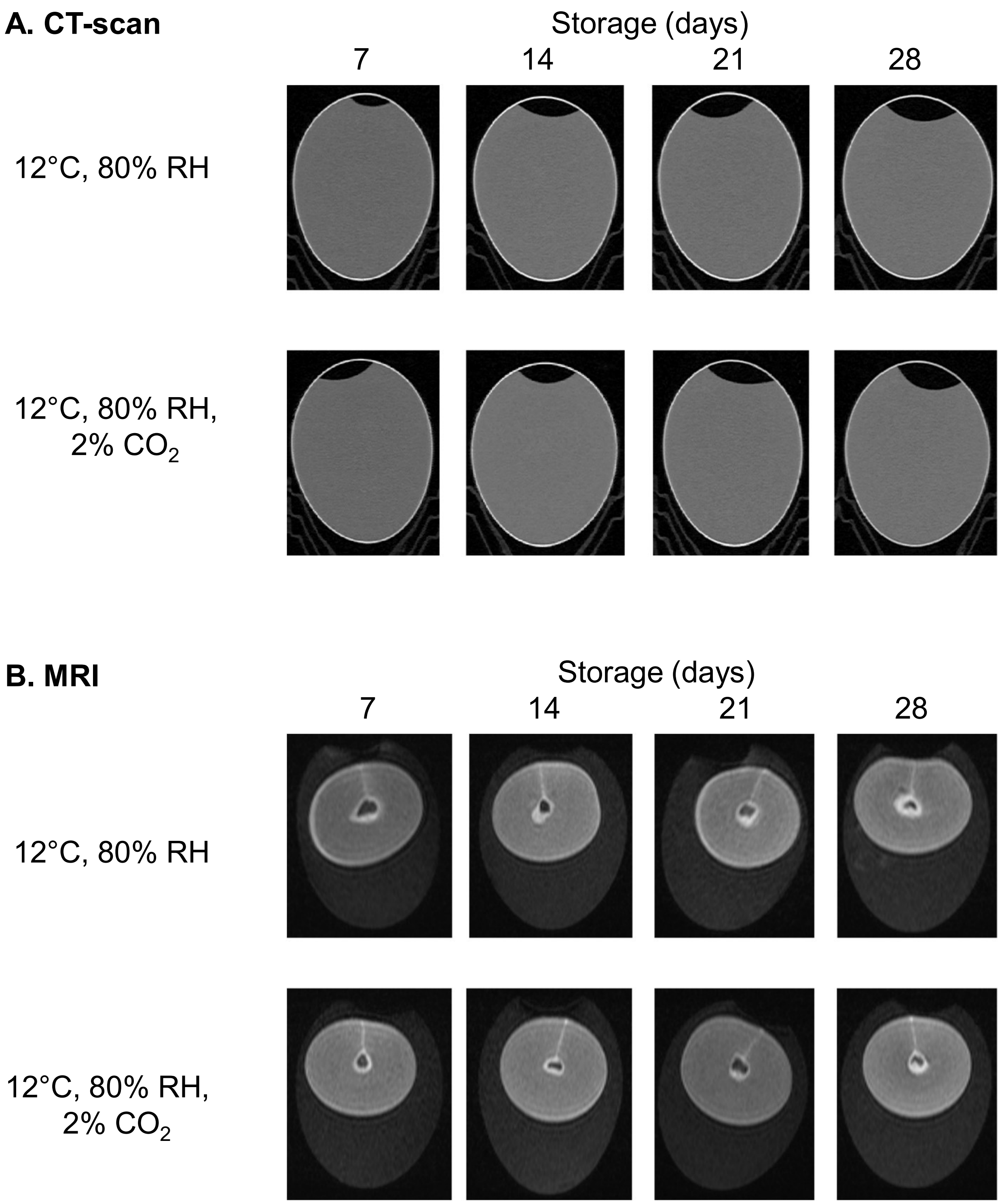

Supplementary data S1

Representative CT scan (A) and MRI (B) images of one egg from each storage × condition batch. For the MRI images, the selected example is not fully representative of the corresponding batch because of the high variability observed among eggs. This figure is intended to illustrate the imaging data obtained rather than to provide a quantitative comparison.
