# Supplementary material for: Impact of 2% carbon dioxide during storage on egg quality and early embryo development
Source: Poult Sci. 2026 Jul 17;105(11):107466. doi: 10.1016/j.psj.2026.107466 (PMC13453520; doi:10.1016/j.psj.2026.107466)
Supplement: Supplementary file 2 [file mmc2.pdf]

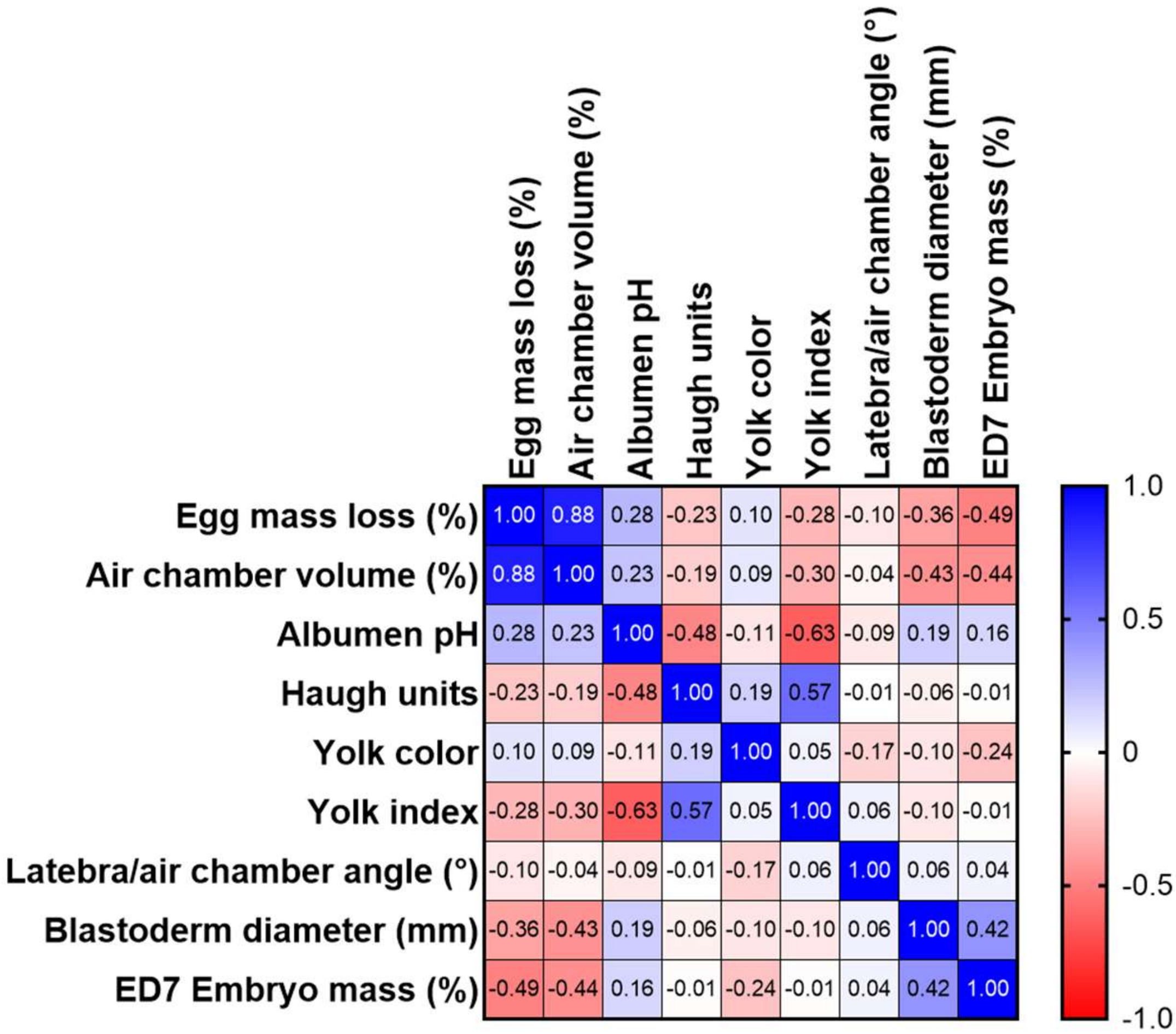

Supplementary data S2

Spearman correlations between embryo relative mass and egg quality parameters
